# Evidence of Infection of Human Embryonic Stem Cells by SARS-CoV-2

**DOI:** 10.3389/fcimb.2022.911313

**Published:** 2022-06-10

**Authors:** Weijie Zeng, Fan Xing, Yanxi Ji, Sidi Yang, Tiefeng Xu, Siyao Huang, Chunmei Li, Junyu Wu, Liu Cao, Deyin Guo

**Affiliations:** Center for Infection and Immunity Study, School of Medicine, Sun Yat-sen University, Shenzhen, China

**Keywords:** SARS-CoV-2, infection, replication, mechanism, hESC

## Abstract

The severe acute respiratory syndrome coronavirus 2 (SARS-CoV-2) was initially described to target the respiratory system and now has been reported to infect a variety of cell types, including cardiomyocytes, neurons, hepatocytes, and gut enterocytes. However, it remains unclear whether the virus can directly infect human embryonic stem cells (hESCs) or early embryos. Herein, we sought to investigate this question in a cell-culture system of hESCs. Both the RNA and S protein of SARS-CoV-2 were detected in the infected hESCs and the formation of syncytium was observed. The increased level of subgenomic viral RNA and the presence of dsRNA indicate active replication of SARS-CoV-2 in hESCs. The increase of viral titers in the supernatants revealed virion release, further indicating the successful life cycle of SARS-CoV-2 in hESCs. Remarkably, immunofluorescence microscopy showed that only a small portion of hESCs were infected, which may reflect low expression of SARS-CoV-2 receptors. By setting |log2 (fold change)| > 0.5 as the threshold, a total of 1,566 genes were differentially expressed in SARS-CoV-2-infected hESCs, among which 17 interferon-stimulated genes (ISGs) were significantly upregulated. Altogether, our results provide novel evidence to support the ability of SARS-CoV-2 to infect and replicate in hESCs.

## Introduction

In January 2020, a novel, severe, acute, respiratory coronavirus 2 (SARS-CoV-2) syndrome pandemic hit the world (Zhou et al., 2020). By May 6, 2022 there were over 513 million people infected with SARS-CoV-2, causing over 6.24 million deaths worldwide (World Health Organization, https://covid19.who.int).

At the beginning of the epidemic, cells with high expression of ACE2 and TMPRSS2 were found to be susceptible to SARS-CoV-2 infection, such as alveolar epithelial type II cells in the lungs and absorptive epithelial cells in the intestine (Ziegler et al., 2020). Later, it was found that other types of cells such as cardiomyocytes, neurons, and hepatocytes could also be infected by SARS-CoV-2 (Wang et al., 2020b; Zhao et al., 2020; Li et al., 2021b; Song et al., 2021). In May 2020, a study reported that SARS-CoV-2 was found in patients’ semen samples (Li et al., 2020a), bringing attentions to the possibility of sexual transmission of SARS-CoV-2 (Sharun et al., 2020; Tatu et al., 2020). To date, there are no documents about SARS-CoV-2 infection in early embryo.

Embryonic stem cell (ESC) determines the embryonic development during early pregnancy and they can differentiate into any cell type of the body. Several types of viruses were found to infect pluripotent stem cells (Bilz et al., 2019; Zahedi-Amiri et al., 2019; Böhnke et al., 2021), which leads to decrease of the pluripotency, triggering autophagy and altering differentiation of stem cells. As the SARS-CoV-2 pandemic continues, the growth and development of the fetus would be severely affected if ESCs can be infected by SARS-CoV-2. However, whether embryos or embryonic stem cells could directly be infected with SARS-CoV-2 remained unclear. Since the establishment of cultured human embryonic stem cell lines such as H1 and H9 (Thomson et al., 1998), the research of human ESCs has been greatly boosted. These cultured human ESC lines can self-renew and differentiate into multiple lineage cell types. This makes it possible to obtain large numbers of hESC in a short period of time instead of preparing a small number of primary hESCs from preimplantation embryos. Thus, it provides convenience for the research of hESC and virus infection.

In this study, we explored the ability of SARS-CoV-2 virus to directly infect human ESCs. We found that H1 and H9 hESCs expressed SARS-CoV-2 viral receptors ACE2 and TMPRSS2 and SARS-CoV-2 could infect these hESCs. We demonstrated the increase of the level of viral RNA and viral protein in hESCs after SARS-CoV-2 infection. The formation of syncytium was observed after 72 hours post-infection (hpi). Moreover, subgenomic viral RNA and dsRNA were detected, and viral titers were increased in the cell culture medium. These results indicated the successful viral replication and viral particle release of SARS-CoV-2 in hESCs. Furthermore, RNA sequencing (RNA-seq) revealed that 17 interferon-stimulated genes (ISGs) were significantly upregulated, including type III interferon gene IFNL1. Taken together, our results provide novel evidence to support the ability of SARS-CoV-2 to infect and replicate in hESCs.

## Materials and Methods

### Cell Culture

Human embryonic stem cell (hESC) H1 and H9 were obtained from Professor Andy Peng Xiang (Center for Stem Cell Biology and Tissue Engineering, Sun Yat-sen University). hESCs were cultured on Matrigel (Corning, 354277) coated plate with daily changed mTeSR Plus media (STEMCELL Technology). hESCs were passed every 4 days with ReleSR (STEMCELL Technology). Caco-2, Calu-3, HUVEC, and BEAS-2B cell lines were obtained from Chinese Academy of Sciences, Shanghai Cell Bank (https://www.cellbank.org.cn). Caco-2 and HUVEC cells were cultured in high glucose Dulbecco’s Modified Eagle’s Medium (DMEM, Gibco), Calu-3 cell was cultured in Minimum Essential Medium (MEM, Gibco), and BEAS-2B cell was cultured in Dulbecco’s Modified Eagle Medium/Nutrient Mixture F-12 (DMEM/F-12, Gibco). Culture media contained 10% fetal bovine serum (FBS, Gibco) and 1% penicillin/streptomycin (Gibco). All cells were cultured in a humidified CO_2_ incubator at 37°C.

### SARS-CoV-2 Virus

SARS-CoV-2 (hCoV-19/CHN/SYSU-IHV/2020 strain, Accession ID on GISAID: EPI_ISL_444969) was isolated from a sputum sample from a woman admitted to the Eighth People’s Hospital of Guangzhou (Ma et al., 2020). The SARS-CoV-2 infection experiments were performed in the BSL-3 laboratory of Sun Yat-sen University.

### Infection of hESCs With SARS-CoV-2

hESCs were seeded in 12-well plates two days before SARS-CoV-2 infection and 2×10^5^ cells (about 40% confluence) were infected at MOI of 0.1 or 0.01 with SARS-CoV-2 for 2 hours at 37°C. Then viral inoculum was removed and the media were replaced by fresh mTeSR Plus for post virus challenge 48 to 72 hours until harvest. At 48 and 72 hpi, supernatants or cells were harvested for Western blot or RT-qPCR analysis.

### Western Blot Analysis

Cells were lysed for 30 min on ice with RIPA buffer and then centrifuged at 16,000g RCF for 15 min at 4°C. Cell lysate was collected and heated with a loading buffer for 10 min at 95°C. Proteins were resolved by SDS-PAGE on a 10% polyacrylamide gel and transferred to 0.45 μm PVDF membrane (Bio-Rad). The membrane was blocked with 5% milk (Solarbio) in TBST (0.05% Tween20) and then probed with a rabbit polyclonal anti-ACE2 antibody (Abcam, ab15348) at 1:1000 dilution or a rabbit monoclonal anti-TMPRSS2 antibody (Abcam, ab109131) or a mouse monoclonal anti-β-actin antibody (Santa Cruz, sc-47778) at 1:1000 dilution or a mouse monoclonal anti-SARS-CoV-2 spike antibody (GeneTex, GTX 632604) overnight at 4°C. The membrane was then incubated with goat-anti-mouse or goat-anti-rabbit secondary antibody conjugated to horseradish peroxidase (Life Technologies, 1:5000) at room temperature for 1 hour. Specific protein bands on the membrane were detected by the ECL detection reagent (Bio-Rad, 1705060) and visualized on a Tanon-5200 Chemiluminescent Imaging System (Tanon Science and Technology, Shanghai, China).

### RNA Extraction and RT-qPCR

For detecting the viral load in the supernatant, SARS-CoV-2 RNA was isolated by Magbead Viral DNA/RNA Kit (CWBIO), and SARS-CoV-2 nucleic acid detection kit (Da an Gene Co., Ltd.) was used to quantify viral RNA. For detecting viral RNA or other gene expression in cells, the total RNA was extracted with TRIzol reagent (Thermo Fisher, USA) and reverse-transcribed into cDNA using PrimeScript RT Master Mix (Takara, RR036A) according to the manufacturer’s instructions. RT-qPCR was performed with PowerUp SYBR Green Master Mix (Thermo Fisher, A25742) on the ABI QuantStudio5 (Applied Biosystems, USA). The relative abundance of target RNA was normalized to the human housekeeping gene actin beta *ACTB*. The primer sequences for detecting SARS-CoV-2 genome or other genes were shown in **Supplementary Material Table S1**.

### RNA Sequencing

After 48 hours post SARS-CoV-2 infection, all cells were harvested for the cDNA library construction in the infection samples. The generation and sequencing of cDNA libraries were done on Illumina Hiseq-2500 platform to generate 150bp PE reads. Raw RNA-seq reads were trimmed using cutadapt (v1.13) of adaptor sequences AGATCGGAAGAGCACACGTCTGACTCCAG, AGATCGGAAGAGCGTCGTGTAGGGAAAGAG, and mapped to human genome (hg38) using STAR (v2.5.3a) with GENCODE (vM18) gene annotations. The number of reads mapping to each gene were calculated using HTSeq (v0.11.2). Differential gene transcriptions were analyzed using DESeq2 (v1.18.1) with |log2 (fold change)| > 0.5.

### Immunofluorescent Staining and TUNEL Fluorescence Assay

hESCs were seeded onto the Matrigel coated coverslips two days before infected with SARS-CoV-2 for 48 hours. Cells were fixed with 4% paraformaldehyde (PFA) in BSL-3 laboratory at room temperature for 3 days and permeabilized by 0.2% Triton X-100 for 20 min. After blocking with 5% bovine serum albumin (BSA) (Abcone, B24726) for 30 min, cells were incubated overnight at 4°C with primary antibodies: mouse monoclonal anti-SARS-CoV-2 Spike antibody (GeneTex, GTX632604), rabbit polyclonal anti-SARS-CoV-2 Spike antibody (GeneTex, GTX135356), rabbit monoclonal anti-Nanog antibody (Cell Signaling Technology, 4903), rabbit monoclonal anti-Oct-4A antibody (Cell Signaling Technology, 2840), mouse monoclonal anti-double-stranded RNA (J2) antibody (SCICONS, 10010500). After the incubation, cells were washed three times with PBS and incubated with Alexa Fluor 488- or 555- or 647- conjugated secondary antibodies for 1 hour at room temperature. For multi-color TUNEL fluorescence assay, positive cells were first detected with CL488 TUNEL fluorescence assay kit (Proteintech, USA PF00006) according to the manufacturer’s instructions and followed with immunofluorescent staining. The nuclei were stained with DAPI (1:10000 diluted in PBS). Cells were washed three times with PBS and mounted on a clean glass slide with Fluoromount-G mounting media (SouthernBiotech, USA). The slides were imaged under fluorescence microscope (Observer Z1, Zeiss) with ZEN microscope software (Zeiss) or confocal microscope (C2, Nikon) with NIS-elements software (Nikon).

### Statistical Analysis

All images were processed and analyzed with ImageJ software (v1.52p). Data were displayed as the means ± standard deviation (± SD), and histograms were generated by GraphPad Prism 8.2.1. Unpaired *t*-test analysis was performed using GraphPad Prism 8.2.1 to evaluate significant differences between two groups being compared and p < 0.05 was considered statistically significant.

## Results

### SARS-CoV-2 Virus Successfully Infects hESCs

**Figure 1:**
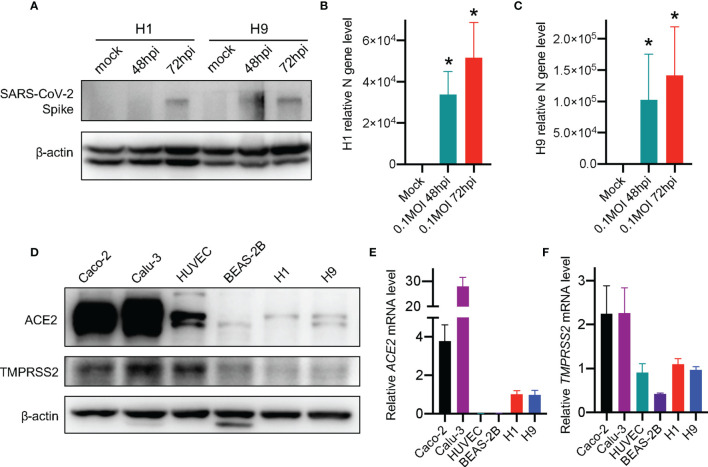
SARS-CoV-2 virus infects hESCs. **(A)** Western blot detection of SARS-CoV-2 spike protein in hESCs after infection. **(B, C)** RT-qPCR detection of SARS-CoV-2 viral RNA in hESCs. Error bars indicate standard deviations of each group. **(D)** Western blot detection of the expression of ACE2 and TMPRSS2 in different cell lines. **(E)** RT-qPCR detection of *ACE2* mRNAs in different human cell lines. **(F)** RT-qPCR detection of *TMPRSS2* mRNAs in different human cell lines. The graphs represent means ± SD from three independent replicates. *p < 0.05.

To identify whether SARS-CoV-2 could infect hESCs, we challenged hESCs with SARS-CoV-2 virus at MOI of 0.1. We found that SARS-CoV-2 could directly infect both H1 and H9 hESCs (**Figure 1A**). The SARS-CoV-2 viral spike protein was detected in the cell lysates of both H1 and H9 hESCs at 72 hours post-infection (hpi). Moreover, the increase of viral RNA was also detected in H1 and H9 hESCs at 48 and 72 hpi (**Figures 1B, C**). The expression of ACE2 and TMPRSS2 is regarded as an important factor for SARS-CoV-2 infection (Chen et al., 2021). Thus, previous studies usually used ACE2 and TMPRSS2-expressing cell lines such as Caco-2 or Calu-3 for SARS-CoV-2 research (Yamamoto et al., 2020; Zecha et al., 2020). We have routinely used Caco-2 and Calu-3 cells to study SARS-CoV-2 infection and anti-SARS-CoV-2 drug development (Li et al., 2022). In this work, we evaluated the expression level of ACE2 and TMPRSS2 in hESCs to explore the pathway for SARS-CoV-2 infection. We found that H1 and H9 hESCs expressed a low level of ACE2 and TMPRSS2 compared to SARS-CoV-2-sensitive human differentiated cell lines (**Figure 1D**). The mRNAs of *ACE2* and *TMPRSS2* were also detected in H1 and H9 hESCs (**Figures 1E, F**). Thus, our results revealed that hESC could be infected with the SARS-CoV-2 virus through the ACE2/TMPRSS2 pathway. However, we found that the ACE2 and TMPRSS2 protein content was not correlated with their gene expression. This phenomenon had also been reported in previous studies and was related to the difference of cell types and cell ages (Bilinska et al., 2020; Qiao et al., 2020). Together, our findings showed that SARS-CoV-2 virus was able to infect human embryonic stem cells.

### The Infection of SARS-CoV-2 Leads to the Formation of Syncytium in hESCs

**Figure 2:**
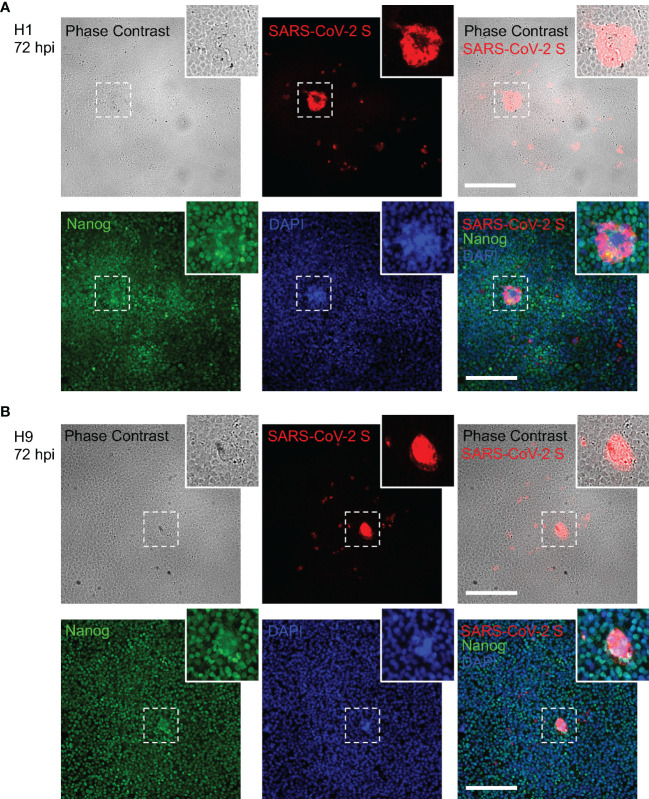
Syncytium formation in hESCs after SARS-CoV-2 infection. **(A)** Combined bright field phase contrast and fluorescence images of H1 hESC after SARS-CoV-2 at 72 hpi. **(B)** Combined bright field phase contrast and fluorescence images of H9 hESC after SARS-CoV-2 at 72 hpi. The inset at the higher-right corner of each image is the enlarged image of the area bordered by the dashed line. Scale bars are 200 μm.

It has been reported that cells infected with SARS-CoV-2 can form cell syncytia (Buchrieser et al., 2020; Cheng et al., 2020). Herein, syncytium was observed in the SARS-CoV-2-infected H1 and H9 hESCs (**Figures 2A, B**). We performed immunofluorescence microscopy and observed large, fused cells under a bright field which were co-located with the SARS-CoV-2 spike protein. H1 and H9 hESCs could form colonies in a normal culture condition (**Figures S1A, B**) and the colonies joined together to form large colonies at 72 hpi (**Figure 2** displayed). These colonies were not impaired by SARS-CoV-2 infection and did not display morphological abnormalities.

In addition, multiple Nanog staining for stem cell marker and DAPI for nucleus were observed in the fused cells. It provided evidence that syncytia were formed in both H1 and H9 hESCs (**Figures 2A, B**). This observation further revealed that the human embryonic stem cell could be infected by SARS-CoV-2. However, immunofluorescence microscopy showed that only a small portion of hESCs were infected and the infection rate of SARS-CoV-2 in H1 and H9 hESCs was 7.68‰ and 8.24‰, respectively (**Table S2**), consistent with the relatively low expression levels of ACE2 and TMPRSS2 in hESCs (**Figure 1D**).

### The Successful Viral Replication and Viral Particle Release in SARS-CoV-2-Infected hESCs

**Figure 3:**
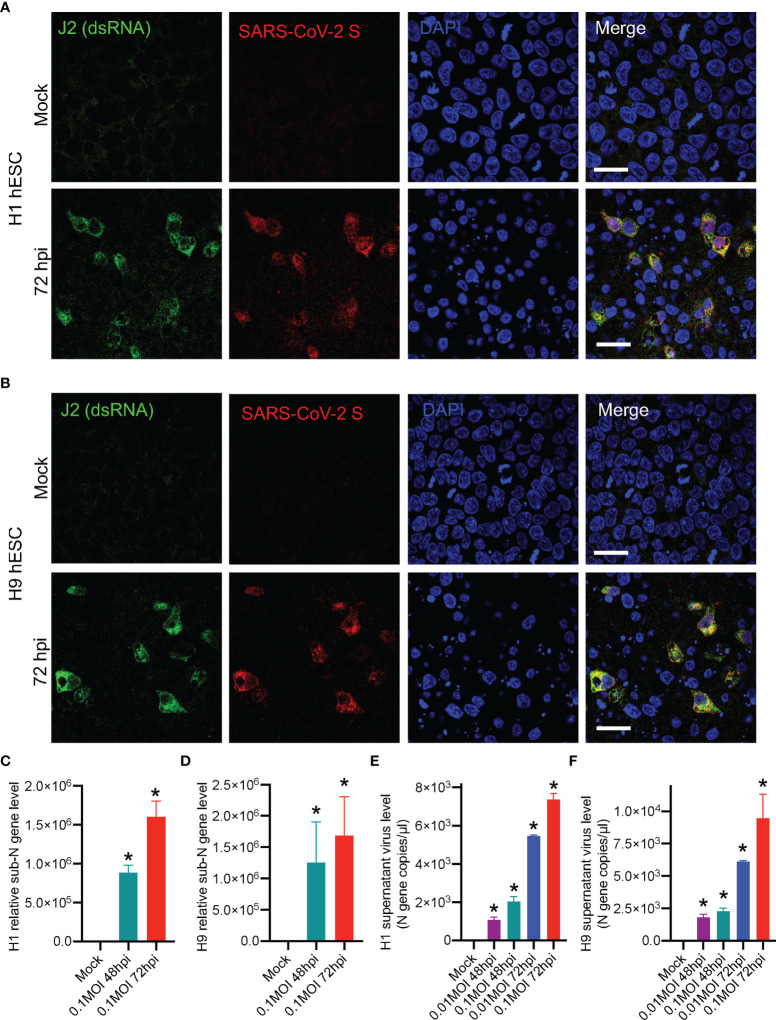
Identifying SARS-CoV-2 replication and viral particle release in hESCs. **(A)** Fluorescence images of H1 hESC after SARS-CoV-2 72 hpi. **(B)** Fluorescence images of H9 hESC after SARS-CoV-2 72 hpi. **(C, D)** RT-qPCR detection of SARS-CoV-2 subgenomic RNA in hESCs. **(E, F)** Detection of SARS-CoV-2 viral RNA in the supernatant by using SARS-CoV-2 nucleic acid detection kit. Error bars indicate standard deviations of each group. *p < 0.05 in specific groups vs. Mock group with *t*-test. All scale bars are 25 μm.

Whether SARS-CoV-2 can replicate after an infection and produce progeny virus is an important part of the virus life cycle. It has been reported that the stem cells were highly resistant to viral infections (Wu et al., 2018) and this demonstrated that SARS-CoV-2 virus was able to infect hESCs (**Figures 1**, **2**). Therefore, we further studied whether SARS-CoV-2 virus could replicate and produce progeny virus in the hESCs. As SARS-CoV-2 is an RNA virus, formation of viral double-stranded RNA (dsRNA) represents an indicator of the viral replication (Klein et al., 2020; Li et al., 2021b). We performed immunofluorescence staining of dsRNA with dsRNA-specific antibody and the dsRNA signal was readily detected in the H1 and H9 hESCs infected by SARS-CoV-2 (**Figures 3A, B**). The subgenomic viral RNA, a unique indicator of coronavirus replication, was also detected (**Figures 3C, D**), suggesting that a successful viral replication took place in the infected hESC. More importantly, the virus titer in the supernatant was increased, indicating the successful viral particle release (**Figures 3E, F**). We used Calu-3 and Caco-2 cells as positive infection controls to evaluate SARS-CoV-2 infectivity and a high virus level was detected in the supernatant of Calu-3 and Caco-2 cells (**Figures S2A, B**), further proving that our SARS-CoV-2 infection system was well operated. These results further demonstrated that the SARS-CoV-2 virus could not only enter but also replicate in human embryonic stem cells.

### SARS-CoV-2 Infection Alters the hESC Transcriptomics

**Figure 4:**
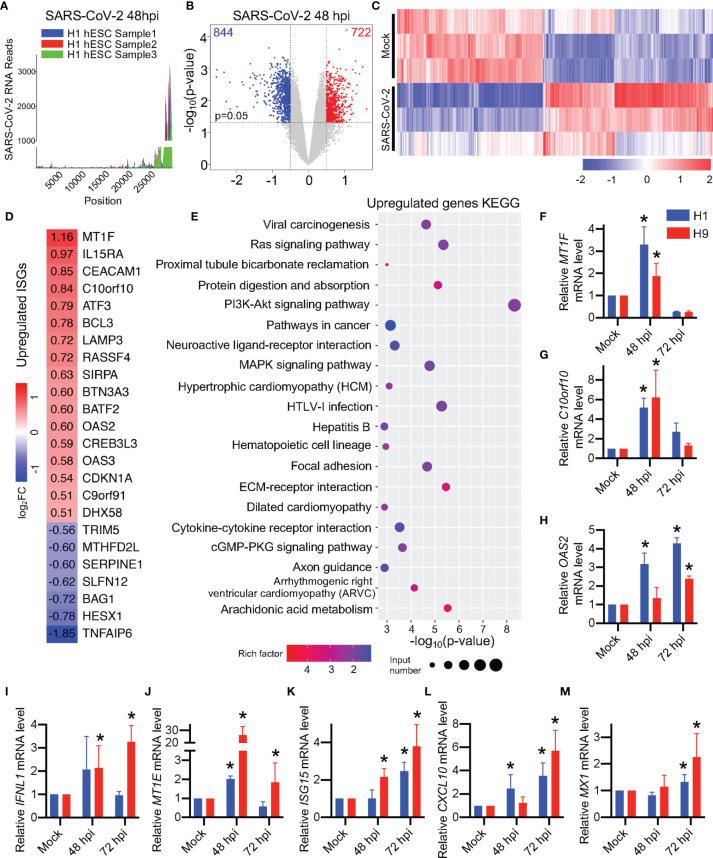
Differentiated expression of transcriptomes in SARS-CoV-2-infected hESCs. **(A)** SARS-CoV-2 RNA reads in RNA-seq analysis. **(B)** Volcano plots showing the expression fold changes and the significance of differentially expressed genes in H1 hESC at 48 hpi (Log_2_FC > 0.5, p-value < 0.05). **(C)** The heatmap of the whole transcriptomes of differentially expressed genes. **(D)** The heatmap of differentially expressed ISGs. **(E)** KEGG analysis of the whole transcriptome of differentially expressed genes. **(F-M)** RT-qPCR detection of ISGs in SARS-CoV-2 infected hESCs. Error bars indicate standard deviations of each group. *p < 0.05 in specific groups vs. Mock group with *t*-test.

We next performed RNA sequencing (RNAseq) to explore the change of hESC transcriptomes after SARS-CoV-2 infection. SARS-CoV-2 RNA reads were detected in RNAseq data (**Figures 4A**), indicating the successful infection of SARS-CoV-2 virus in H1 hESCs. These reads did not show uniform distribution but were enriched at the 3’-end of SARS-CoV-2 genome (**Figure 4A**). RNAseq transcriptome analyses detected expression changes in 1,566 of the 39,219 genes in SARS-CoV-2-infected H1 hESC at 48 hpi (**Figures 4B, C**), with 844 genes downregulated and 722 genes upregulated (log_2_FC >0.5 was considered an upregulated gene and log_2_FC <-0.5 was considered a downregulated gene, see **Supplementary DEG.xlsx File**). We found a set of interferon stimulated genes (ISGs) that were expressed in H1 hESCs (**Figure 4C**, and **Supplementary DEG.xlsx File**), which was consistent with previous research that shows hESCs expressed a subset of intrinsic ISGs (Wu et al., 2018). Among these differentially expressed genes, 17 ISGs were upregulated and 7 were downregulated significantly (**Figure 4D**). The Kyoto Encyclopedia of Genes and Genomes (KEGG) pathways enriched in SARS-CoV-2 48 hpi H1 hESCs were assessed. In KEGG, innate immune response and cell proliferation, differentiation, apoptosis, and migration regulatory function were observed (**Figure 4E**). We chose three of these 17 upregulated ISGs to confirm the RNAseq result by RT-qPCR (**Figures 4F–H**). The expression level of MT1F, C10orf10, and OAS2 gene was significantly upregulated (**Figures 4F–H**).

Pluripotent stem cells were widely considered as IFN pathway-defective during viral infection and responded weakly to IFN treatment (Hong and Carmichael, 2013). However, in a previous study, type III interferon IFNL1 was found to be upregulated after RNA virus infection in pluripotent stem cells (Bilz et al., 2019). Interestingly, we found a few reads of IFNL1 in RNAseq (about 1.6 RPKM) but there were no significant changes and the number of the reads was higher compared with type I interferon IFNB and IFNA reads (0 RPKM) in H1 hESC (**Figure 4C** and **Table S2**). Nevertheless, although the read levels in the RNAseq was low, we found that the type III interferon gene IFNL1 was upregulated after SARS-CoV-2 infection in H9 hESC by RT-qPCR (**Figure 4I**) and the downstream ISGs of IFNL1 were upregulated (**Figures 4J–M**). However, there were no significant changes of type I interferon genes in hESCs during SARS-CoV-2 infection (**Figure S3**). It was consistent with the previous report that the type I interferon pathway in hESC did not respond during RNA virus infection (Burke et al., 1978; Földes et al., 2010; Guo et al., 2015; Guo, 2017; Bilz et al., 2019). Our results show that the SARS-CoV-2 infection alters the hESC transcriptomes and the type III interferon pathway may be involved.

### SARS-CoV-2 Infection Alters the hESC Viability and Pluripotency

**Figure 5:**
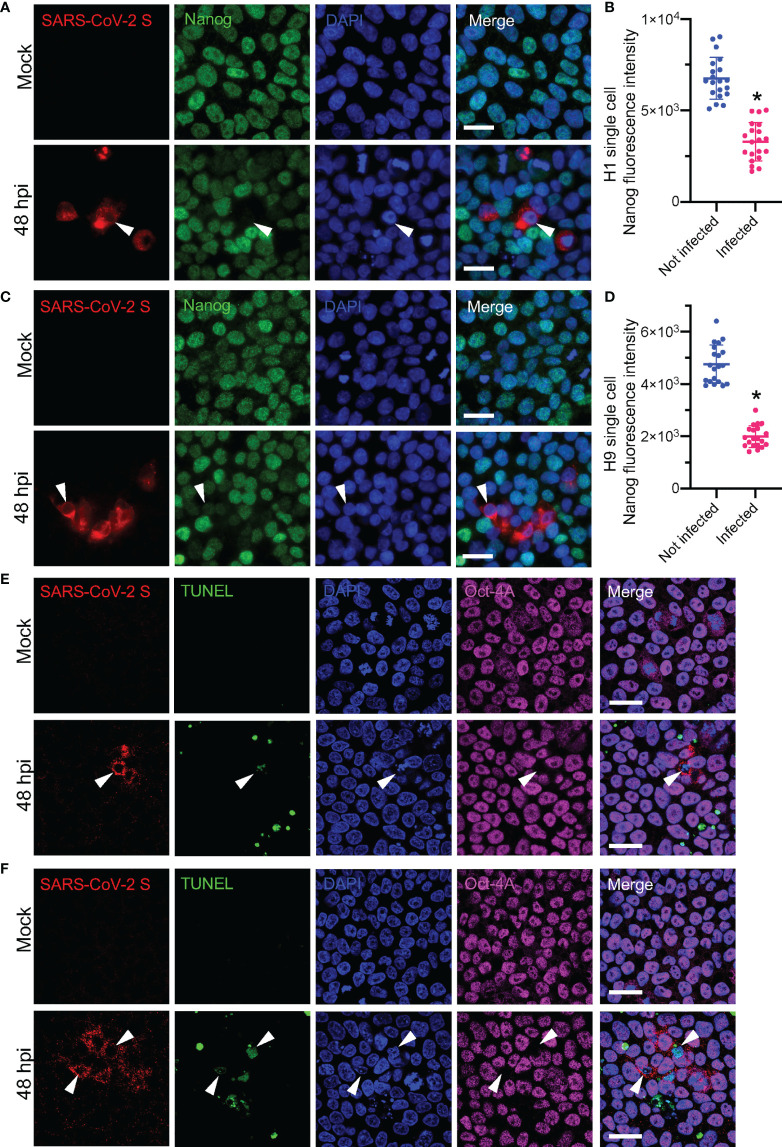
Detecting apoptosis and stem cell pluripotency markers in SARS-CoV-2-infected hESCs. **(A)** Fluorescence images of H1 hESC after SARS-CoV-2 48 hpi. **(B)** Plots of Nanog fluorescence intensities of single cells of H1 hESC, related to **(A)**. **(C)** Fluorescence images of H9 hESC after SARS-CoV-2 48 hpi. **(D)** Plots of Nanog fluorescence intensities of H9 hESC single cells, related to **(C)**. **(E)** Multi-color TUNEL fluorescence assay of H1 hESC after SARS-CoV-2 48 hpi. **(F)** Multi-color TUNEL fluorescence assay of H9 hESC after SARS-CoV-2 48 hpi. White arrowhead indicated the pluripotency decreased SARS-CoV-2 infected hESC. Error bars indicate standard deviations of each group. *p < 0.05 in infected group vs. not infected group with *t*-test. All scale bars are 25 μm.

In human embryonic stem cells, transcription factors such as Nanog and Oct-4 are connected with other factors to maintain hESC pluripotency (Heurtier et al., 2019). In a previous study, the human-induced pluripotent stem cell (hiPSC) was reported to have decreased viability and pluripotency after an influenza A virus infection (Zahedi-Amiri et al., 2019). In this study, we found the H1 and H9 hESCs pluripotency marker Nanog was decreased after SARS-CoV-2 infection (**Figures 5A, B**, indicated with white arrowhead). Recently, it has been demonstrated that the SARS-CoV-2 viral spike protein can induce apoptosis (Li et al., 2021a). Accordingly, we speculated that the viability may be affected in the pluripotency-decreasing hESCs. To evaluate possible apoptosis of the infected hESCs, we performed multi-color terminal deoxynucleotidyl transferase dUTP nick end labeling (TUNEL) in the SARS-CoV-2-infected H1 and H9 hESCs. We found that TUNEL-positive cells were significantly increased after SARS-CoV-2 infection (**Figures 5E, F**). In the SARS-CoV-2-infected cells, we confirmed that TUNEL-positive cells were accompanied by a decrease in Oct-4 (**Figures 5E, F**, indicated with white arrowhead). In addition, H1 hESC RNA-seq results showed that there were several genes with altered expression levels associated with the apoptosis (see **Supplementary DEG.xlsx File**). These genes include AATK (Apoptosis Associated Tyrosine Kinase), HRK (Harakiri, BCL2 Interacting Protein), PPP1R13B (Protein Phosphatase 1 Regulatory Subunit 13B), TMBIM1 (Transmembrane BAX Inhibitor Motif Containing 1), FAIM (Fas Apoptotic Inhibitory Molecule), TIA1 (TIA1 Cytotoxic Granule Associated RNA Binding Protein), CYCS (Cytochrome C, Somatic) and CASP3 (Caspase 3). In conclusion, SARS-CoV-2 infection triggered the apoptosis of hESC, the decrease in cell viability, and pluripotency.

## Discussion

Previously, human induced pluripotent stem cells overexpressing the SARS-CoV-2 viral receptor ACE2 (ACE2-iPSCs) or differentiated lineage cells derived from iPSCs were used in a SARS-CoV-2 study (Zhang et al., 2020; Sano et al., 2021). We are interested in whether hESC itself can be infected by SARS-CoV-2. In this study, we revealed that SARS-CoV-2 virus could directly infect hESC. Sano et al. reported that SARS-CoV-2 does not infect undifferentiated human iPSCs (Sano et al., 2021), the discrepancy between iPSC and hESCs needs to be further investigated. We found that hESCs expressed a low level of viral receptors ACE2 and TMPRSS2 but they still can contribute to the SARS-CoV-2 infection. Consequently, a small portion of hESCs were infected and the SARS-CoV-2 virus was reproduced in the infected hESC. However, the low infection rate of SARS-CoV-2 in hESC (**Figure 2**) suggested that only a small part of hESC could be infected, which was consistent with low ACE2 and TMPRSS2 expression in hESC (**Figure 1A**). These results suggest that ACE2-iPS cells or iPSC-derived cells or organoids might be more suitable for SARS-CoV-2 screening than hESC. Recently, CD147, ASGR1, KREMEN1, and AXL have been reported to function as potential receptors (Wang et al., 2020a; Wang et al., 2021; Gu et al., 2022). A few years ago, low expression level of CD147 was found in KhES-1 hESC cell line (Higashi et al., 2015). Therefore, our current study did not define which SARS-CoV-2 receptors mediated the viral infection in hESCs. Whether ACE2 was the main receptor that mediated SARS-CoV-2 infection in hESC needs to be further studied in the future.

SARS-CoV-2 was found to be mainly transmitted by air droplets (Li et al., 2020b) while other routes of transmission were reported. Pregnant women infected with SARS-CoV-2 could transmit the virus to their fetuses (Alzamora et al., 2020; Dong et al., 2020; Fenizia et al., 2020). However, vertical transmission was not reported in SARS-CoV and Middle East respiratory syndrome coronavirus (MERS-CoV) infection. The potential for the vertical transmission of SARS-CoV-2 needs to be carefully investigated. Our results show the SARS-CoV-2 virus could replicate and trigger apoptosis in the infected hESC (**Figures 3**, **5**) which could be fatal to the pregnant fetus.

It has been reported that embryonic stem cells were highly resistant to virus infection (Wu et al., 2019). Several antiviral mechanisms gave stem cells protection against viral infection (Wolf and Goff, 2009; Wu et al., 2018; Poirier et al., 2021; Wu et al., 2021). However, the antiviral resistance of stem cells is not effective against all viral infections. Unlike differentiated somatic cells, stem cells do not rely on the interferon pathway to exert antiviral effects and do not produce type I interferon during viral infection (Guo et al., 2015; Guo, 2017; Wu et al., 2019). In our study, we found that the type I interferon pathway was not activated in hESC with SARS-CoV-2 infection, and IFN-β, IL-1β, and IL-6 were not significantly increased (**Figure S2**). Other researchers also found that human iPSCs infected with the rubella virus exhibited an attenuated type I interferon response (Bilz et al., 2019).

However, a few of ISGs of hESCs were up-regulated (**Figures 4F–M**), indicating that hESCs may exhibit immune response in the SARS-CoV-2 infection. Previous studies have found that some RNA virus infections triggered the type III interferon pathway response in iPSCs (Bilz et al., 2019; Zahedi-Amiri et al., 2019). In this study, we also observed an upregulation of the type III interferon pathway IFNL1 (**Figure 4I**). However, only a few ISGs were altered in hESCs throughout the SARS-CoV-2 infection and we thought that it was unlikely that hESCs relied on the type III interferon pathway response to defend against SARS-CoV-2 infection. Some researchers have found that overexpression of some ISGs in hESCs can alter the differentiation of stem cells (He et al., 2020). It is reasonable to speculate that the type III interferon response of hESCs may relate to the regulation of stem cell differentiation rather than antiviral effects, which needs to be further studied.

Stem cells are found to be weakly responsive to interferon and usually have little change in ISG levels (Wu et al., 2018; Wu et al., 2019). Our RNA-seq results showed that a total of 17 ISGs were up-regulated in H1 hESC during SARS-CoV-2 infection. Among them, the highest log_2_FC value was MT1F, which reached 1.16 (about 2.23 times upregulation), and the log_2_FC values ​​of the remainder of the up-regulated ISGs were between 0.5 and 1.0, indicating little change in the ISG levels of H1 hESC. When other stem cells such as hiPSCs were infected with the rubella virus, a small number of ISGs such as MX1, IFIT1, IFITM1, IFITM3, ISG15, IRF9, and STAT1 were also up-regulated but IFIT1 with the highest log_10_FC value was only 0.6 (about 3.98 times upregulation) (Bilz et al., 2019). These results indicate that the change in ISGs caused by the SARS-CoV-2 or other RNA virus infection of hESC were not dramatic. This is different from the differentiated somatic cells which may produce robust interferon responses during virus infection.

Studies have shown that SARS-CoV-2 could infect macrophages, monocytes, and T cells but only progressed to an abortive infection (Abdelmoaty et al., 2021; Swadling et al., 2022). Although we found only a small portion of hESCs were infected, the formation of viral subgenomic viral RNA and dsRNA, as well as the increase in SARS-CoV-2 titer in culture medium, demonstrated that the SARS-CoV-2 infection in hESC should be a productive infection.

## Data Availability Statement

The datasets presented in this study can be found in online repositories. The RNAseq raw reads after SARS-CoV-2 infection in H1 hESC can be found on SRA database (PRJNA817715, https://www.ncbi.nlm.nih.gov/sra/PRJNA817715). The names of the repository/repositories and accession number(s) can be found in the article/**Supplementary Material**.

## Author Contributions

DG conceived and supervised the study; WZ, JW, and DG designed the experiments, analyzed the data and wrote the manuscript. WZ, FX, and LC conducted all the major experiments. YJ, SY, TX, SH, and CL participated in some of the experiments or helped with reagents and discussions. All authors contributed to the article and approved the submitted version.

## Funding

The work is supported by the National Natural Science Foundation of China (#81620108020 and #32041002 to DG; #31800151 to JW; #81803568 to FX), Shenzhen Science and Technology Program (#KQTD20180411143323605 and #JSGG20200225150431472 to DG; #JCYJ20170818162249554 to FX; #GXWD20201231165807008, 20200825183117001 to JW). DG is also supported by National Ten-thousand Talents Program and Guangdong Zhujiang Talents Program (2016LJ06Y540).

## Conflict of Interest

The authors declare that the research was conducted in the absence of any commercial or financial relationships that could be construed as a potential conflict of interest.

## Publisher’s Note

All claims expressed in this article are solely those of the authors and do not necessarily represent those of their affiliated organizations, or those of the publisher, the editors and the reviewers. Any product that may be evaluated in this article, or claim that may be made by its manufacturer, is not guaranteed or endorsed by the publisher.

## Supplementary Material

Supplementary Figure 1 Morphology of hESC colony. **(A)** Bright field phase contrast image of H1 hESC in normal culture condition before SARS-CoV-2 infection. **(B)** Bright field phase contrast image of H9 hESC in normal culture condition before SARS-CoV-2 infection. Scale bars are 200 μm.

Supplementary Figure 2 Detection of SARS-CoV-2 viral RNA in supernatant by using SARS-CoV-2 nucleic acid detection kit. **(A)** Calu-3 cell virus level in the supernatant after 48 hpi of SARS-CoV-2. **(B)** Caco-2 cell virus level in the supernatant after 48 hpi of SARS-CoV-2. Error bars indicate standard deviations of each group. *p < 0.05 in 0.05MOI 48hpi group vs. Mock group with *t*-test.

Supplementary Figure 3 Not significantly changed ISGs in hESCs after SARS-CoV-2 infection. (**A-C**) RT-qPCR detection of ISGs in SARS-CoV-2 infected hESCs. Error bars indicate standard deviations of each group.
