# Supplementary figures and images for: Evidence of Infection of Human Embryonic Stem Cells by SARS-CoV-2
Source: Front Cell Infect Microbiol. 2022 Jun 10;12:911313. doi: 10.3389/fcimb.2022.911313 (PMC9226488; doi:10.3389/fcimb.2022.911313)

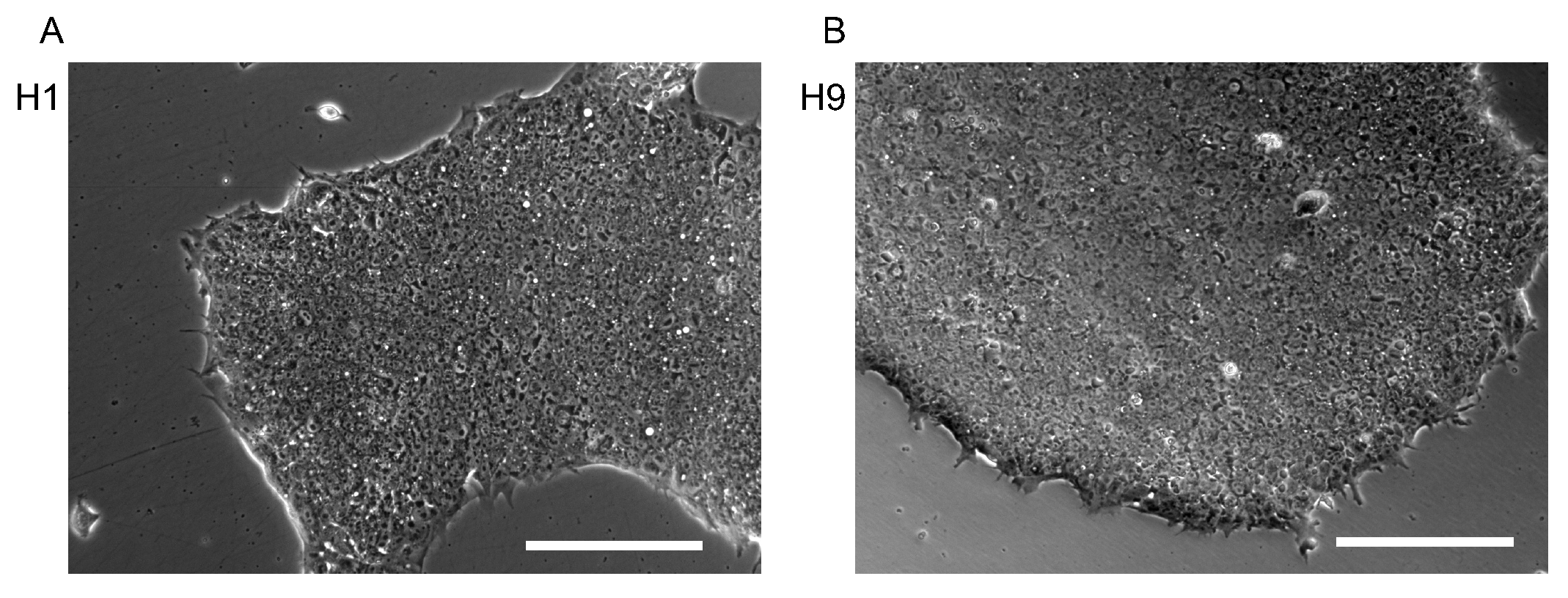

Supplement: Supplementary Figure 1 — Morphology of hESC colony. (A) Bright field phase contrast image of H1 hESC in normal culture condition before SARS-CoV-2 infection. (B) Bright field phase contrast image of H9 hESC in normal culture condition before SARS-CoV-2 infection. Scale bars are 200 μm. [file Image_1.tif]

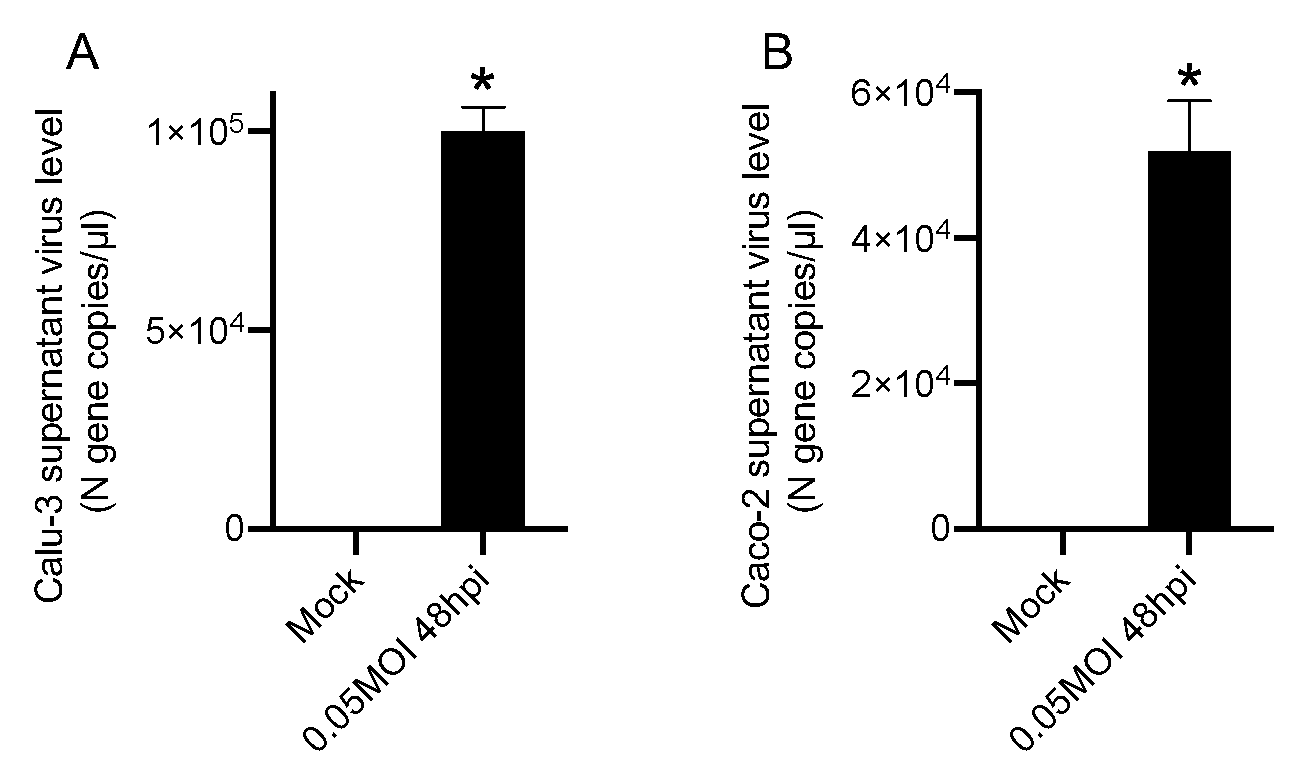

Supplement: Supplementary Figure 2 — Detection of SARS-CoV-2 viral RNA in supernatant by using SARS-CoV-2 nucleic acid detection kit. (A) Calu-3 cell virus level in the supernatant after 48 hpi of SARS-CoV-2. (B) Caco-2 cell virus level in the supernatant after 48 hpi of SARS-CoV-2. Error bars indicate standard deviations of each group. *p < 0.05 in 0.05MOI 48hpi group vs. Mock group with t-test. [file Image_2.tif]

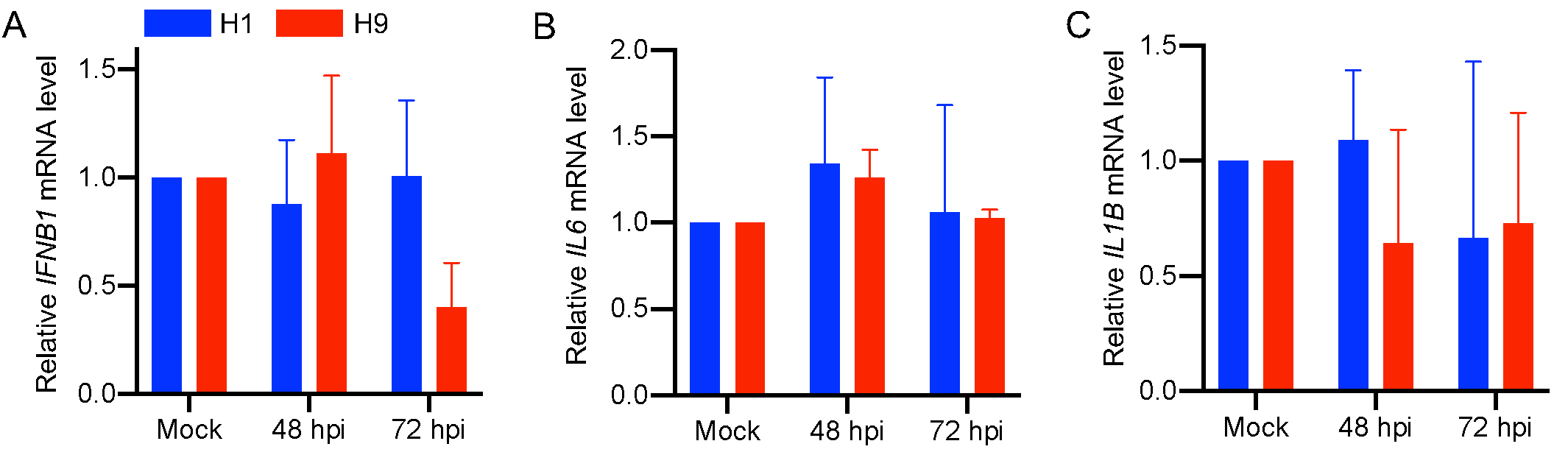

Supplement: Supplementary Figure 3 — Not significantly changed ISGs in hESCs after SARS-CoV-2 infection. (A-C) RT-qPCR detection of ISGs in SARS-CoV-2 infected hESCs. Error bars indicate standard deviations of each group. [file Image_3.tif]
