# Supplementary material for: Evidence of Infection of Human Embryonic Stem Cells by SARS-CoV-2
Source: Front Cell Infect Microbiol. 2022 Jun 10;12:911313. doi: 10.3389/fcimb.2022.911313 (PMC9226488; doi:10.3389/fcimb.2022.911313)
Supplement: Supplementary file 5 [file Table_1.docx]

Table S1 RT-qPCR primers used in this study

| **Gene** | **Forward qPCR primers** | **Reverse qPCR primers** |
| --- | --- | --- |
| *ACTB* | AAGGAGCCCCACGAGAAAAAT | ACCGAACTTGCATTGATTCCAG |
| *ACE2* | CGAAGCCGAAGACCTGTTCTA | GGGCAAGTGTGGACTGTTCC |
| *TMPRSS2* | CAAGTGCTCCAACTCTGGGAT | AACACACCGATTCTCGTCCTC |
| *SARS-CoV-2*  *N gene* | AAGAAATTCAACTCCAGGCAGC | GCTGGTTCAATCTGTCAAGCAG |
| *SARS-CoV-2*  *sub-N gene* | CCAGGTAACAAACCAACAA | TGAGTGAGAGCGGTGAACCAA |
| *MT1F* | TGGACCCCAACTGCTCCT | CAGCAGCTCTTCTTGCAGG |
| *C10orf10* | GTGAGGTCTATATCTCGACTGGC | ACTGAAACGTGCGGTGATGT |
| *OAS2* | AGGTGGCTCCTATGGACGG | TTTATCGAGGATGTCACGTTGG |
| *IFNL1* | CACATTGGCAGGTTCAAATCTCT | CCAGCGGACTCCTTTTTGG |
| *MT1E* | TCAGGTTGGGAGGGAACTCAA | GAAAGCCTGGAGAGGGAATGA |
| *ISG15* | TCCTGGTGAGGAATAACAAGGG | GTCAGCCAGAACAGGTCGTC |
| *CXCL10* | GTGGCATTCAAGGAGTACCTC | TGATGGCCTTCGATTCTGGATT |
| *MX1* | GTTTCCGAAGTGGACATCGCA | CTGCACAGGTTGTTCTCAGC |
| *IFNB1* | CATTACCTGAAGGCCAAGGA | CAATTGTCCAGTCCCAGAGG |
| *IL6* | ACTCACCTCTTCAGAACGAATTG | CCATCTTTGGAAGGTTCAGGTTG |
| *IL1B* | ATGATGGCTTATTACAGTGGCAA | GTCGGAGATTCGTAGCTGGA |
