# Supplementary material for: Evidence of Infection of Human Embryonic Stem Cells by SARS-CoV-2
Source: Front Cell Infect Microbiol. 2022 Jun 10;12:911313. doi: 10.3389/fcimb.2022.911313 (PMC9226488; doi:10.3389/fcimb.2022.911313)
Supplement: Supplementary file 6 [file Table_2.docx]

Table S2 Infection rate of SARS-CoV-2 in hESCs

| **H1 hESC** | | |  | **H9 hESC** | | |
| --- | --- | --- | --- | --- | --- | --- |
| **Infected cell count** | **Total cell count** | **Infection rate (%)** |  | **Infected cell count** | **Total cell count** | **Infection rate (%)** |
| 22 | 3856 | 0.57 |  | 27 | 3808 | 0.71 |
| 20 | 3824 | 0.52 |  | 20 | 3792 | 0.53 |
| 47 | 3880 | 1.21 |  | 41 | 3848 | 1.06 |
| 35 | 3844 | 0.91 |  | 40 | 3812 | 1.05 |
| 24 | 3788 | 0.63 |  | 29 | 3780 | 0.77 |

Cells with positive immunofluorescence staining of SARS-CoV-2 S protein were considered as SARS-CoV-2 infected.
