# Supplementary material for: Evidence of Infection of Human Embryonic Stem Cells by SARS-CoV-2
Source: Front Cell Infect Microbiol. 2022 Jun 10;12:911313. doi: 10.3389/fcimb.2022.911313 (PMC9226488; doi:10.3389/fcimb.2022.911313)
Supplement: Supplementary file 8 [file Presentation_1.pptx]

## Slide 1
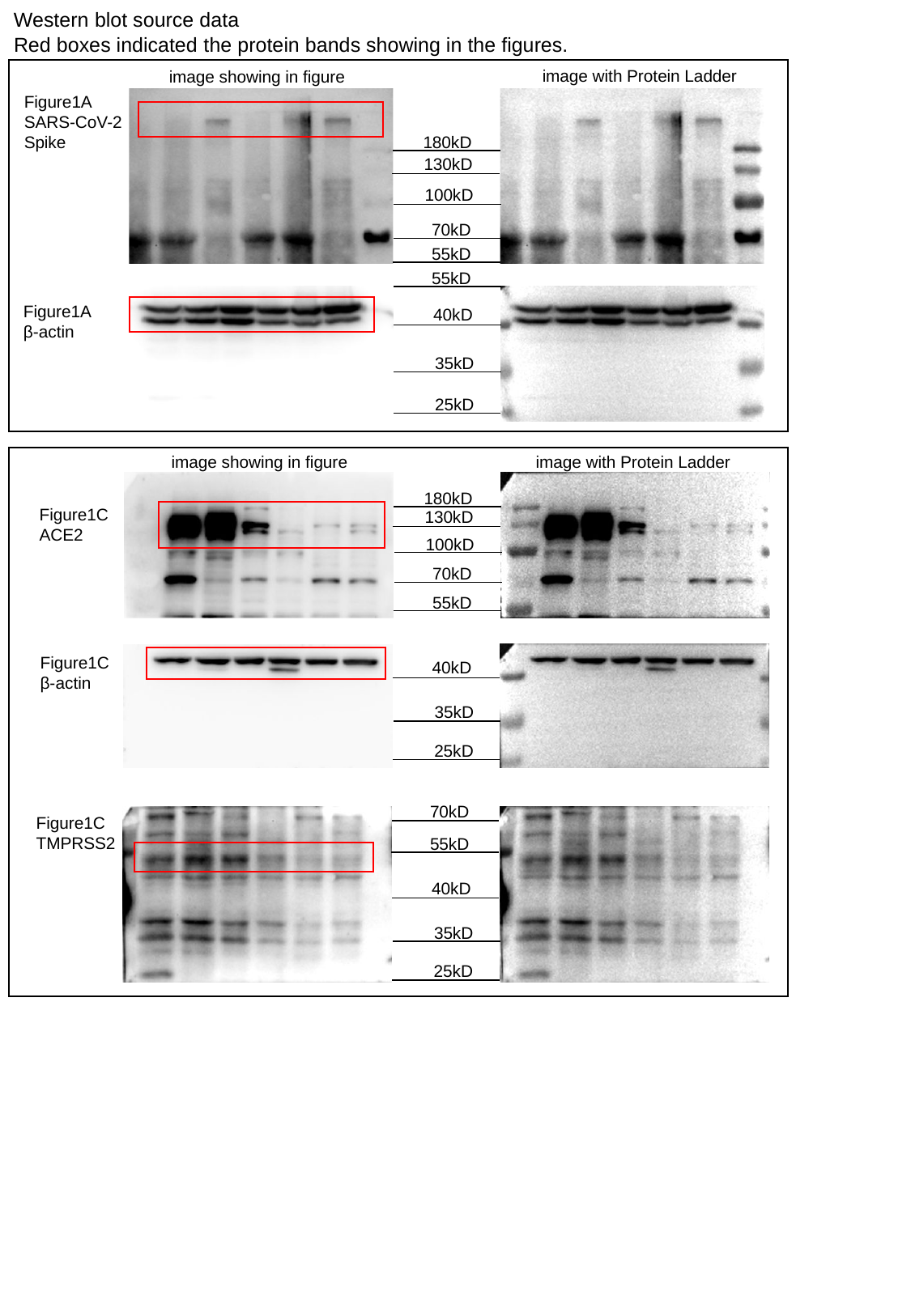

Western blot source data
Red boxes indicated the protein bands showing in the figures.
image with Protein Ladder
image showing in figure
Figure1A
SARS-CoV-2
Spike
180kD
130kD
100kD
70kD
55kD
55kD
Figure1A
β-actin
40kD
35kD
25kD
image with Protein Ladder
image showing in figure
180kD
Figure1C
ACE2
130kD
100kD
70kD
55kD
Figure1C
β-actin
40kD
35kD
25kD
70kD
Figure1C
TMPRSS2
55kD
40kD
35kD
25kD
